# Discovery of potential pathways for biological conversion of poplar wood into lipids by co-fermentation of *Rhodococci* strains

**DOI:** 10.1186/s13068-019-1395-x

**Published:** 2019-03-19

**Authors:** Xiaolu Li, Yucai He, Libing Zhang, Zhangyang Xu, Haoxi Ben, Matthew J. Gaffrey, Yongfu Yang, Shihui Yang, Joshua S. Yuan, Wei-Jun Qian, Bin Yang

**Affiliations:** 1 Bioproducts, Sciences and Engineering Laboratory, Department of Biological Systems Engineering, Washington State University, 2710 Crimson Way, Richland, WA 99354 USA; 2 Biological Sciences Division, Pacific Northwest National Laboratory, Richland, WA 99352 USA; 3 State Key Laboratory of Biocatalysis and Enzyme Engineering, Hubei Collaborative Innovation Center for Green Transformation of Bio-resources, Environmental Microbial Technology Center of Hubei Province, and School of Life Sciences, Hubei University, Wuhan, 430062 China; 4 Department of Plant Pathology and Microbiology, Texas A&M University, College Station, TX 77840 USA

**Keywords:** Lignin, Lipid, *Rhodococcus opacus* PD630, *Rhodococcus jostii* RHA1, Co-fermentation, Proteomics, β-Ketoadipate pathway, Phenylacetic acid (PAA) pathway

## Abstract

**Background:**

Biological routes for utilizing both carbohydrates and lignin are important to reach the ultimate goal of bioconversion of full carbon in biomass into biofuels and biochemicals. Recent biotechnology advances have shown promises toward facilitating biological transformation of lignin into lipids. Natural and engineered *Rhodococcus* strains (e.g., *R. opacus* PD630*, R. jostii* RHA1, and *R. jostii* RHA1 VanA^−^) have been demonstrated to utilize lignin for lipid production, and co-culture of them can promote lipid production from lignin.

**Results:**

In this study, a co-fermentation module of natural and engineered *Rhodococcus* strains with significant improved lignin degradation and/or lipid biosynthesis capacities was established, which enabled simultaneous conversion of glucose, lignin, and its derivatives into lipids. Although *Rhodococci* sp. showed preference to glucose over lignin, nearly half of the lignin was quickly depolymerized to monomers by these strains for cell growth and lipid synthesis after glucose was nearly consumed up. Profiles of metabolites produced by *Rhodococcus* strains growing on different carbon sources (e.g., glucose, alkali lignin, and dilute acid flowthrough-pretreated poplar wood slurry) confirmed lignin conversion during co-fermentation, and indicated novel metabolic capacities and unexplored metabolic pathways in these organisms. Proteome profiles suggested that lignin depolymerization by *Rhodococci* sp. involved multiple peroxidases with accessory oxidases. Besides the β-ketoadipate pathway, the phenylacetic acid (PAA) pathway was another potential route for the in vivo ring cleavage activity. In addition, deficiency of reducing power and cellular oxidative stress probably led to lower lipid production using lignin as the sole carbon source than that using glucose.

**Conclusions:**

This work demonstrated a potential strategy for efficient bioconversion of both lignin and glucose into lipids by co-culture of multiple natural and engineered *Rhodococcus* strains. In addition, the involvement of PAA pathway in lignin degradation can help to further improve lignin utilization, and the combinatory proteomics and bioinformatics strategies used in this study can also be applied into other systems to reveal the metabolic and regulatory pathways for balanced cellular metabolism and to select genetic targets for efficient conversion of both lignin and carbohydrates into biofuels.

**Electronic supplementary material:**

The online version of this article (10.1186/s13068-019-1395-x) contains supplementary material, which is available to authorized users.

## Background

Cellulosic biomass, comprised of about 10–25% lignin, 20–30% hemicellulose, and 40–50% cellulose, is an abundant sustainable resource to support large-scale, low-cost production of transportation fuels [1, 2]. However, a large-scale and robust platform for biomass-derived biofuel is mostly lacking [3]. Current biological processing platforms only convert plant polysaccharides into biofuels, resulting in the formation of a significant process stream rich in lignin. It is then utilized as an energy resource for power/electrical generation, partially due to the lack of efficient chemical conversion processes to convert both sugars and lignin into transportation biofuels or high-value chemicals [4–10]. The utilization of all of carbons from biomass for biofuels and bioproducts production offers a significant opportunity for enhancing the overall operational efficiency and cost competitiveness of a lignocellulosic biorefinery.

Although lignin is more energy dense than cellulose and hemicellulose due to its higher carbon–oxygen ratio [11], it is much more difficult to depolymerize due to its complex molecular structure. Structural heterogeneity also leads to a broad spectrum of breakdown products, substantially compromising the efficiency of chemical catalysis approaches for product synthesis and purification. On the contrary, the microbial conversion of lignin enables targeting heterogeneous lignin to specific value-added products. Compared with fungal systems, the ligninolytic capability of bacteria is less well understood, and thus attracts intensive studies considering the immense biochemical versatility and environmental adaptability of bacteria [8–10, 12–17]. In chemoheterotrophic organisms, triacylglycerides (TAGs) are synthesized by bioconversion of organic compounds (e.g., sugars and organic acids) derived from the lignocellulosic biomass. These TAGs of monoalkyl esters of long-chain fatty acids combined with glycerol can be converted into fatty acid short-chain alcohol esters in the form of FAME (methanol) and FAEE (ethanol) for biodiesel production, which is now well established on a commercial scale [1, 2, 14, 18], but the cost associated with the development of biofuels remains challenging. Several research groups have developed microbial technology that is capable of converting lignin and/or biorefinery wastes into TAGs through the *Rhodococcus* strains [15, 19–22]. However, the routes from lignin to lipid remain unclear.

Several *Rhodococcus* strains possess metabolic pathways for oxidative ring opening of central aromatic intermediates via the β-ketoadipate pathway [3, 14, 23], which enables the shuttling of aromatic-derived carbon into central carbon metabolism via the tricarboxylic acid (TCA) cycle. These pathways contribute to microbial conversion of various lignin-derived aromatic molecules into structure carbon and energy sources [24, 25]. TAG accumulation is a common feature shared by many *Rhodococcus* members as one of the in vivo storage compounds under nitrogen-limited conditions [26]. The omics studies on *R. opacus* PD630 revealed genes enriched in pathways of lipid transportation, synthesis, and metabolism [27], which supported the strain’s accumulation of lipid around 80% of cell dry weight (CDW) [28]. On the other hand, many aromatics funneling pathways were elucidated in *R. opacus* PD630 as well [17, 29]. Many studies have been conducted to evaluate the strains’ growth and lipid yield on lignin from various sources or lignin model compounds. The bioconversion from lignin to lipid by *R. opacus* utilizing ultrasonicated ethanol organosolv lignin with the yield of 0.004 g/g substrate and 4.08% (based on CDW) has been reported in 2013 [1]. The residues from dilute-acid pretreatment mainly consisting of lignin and polysaccharides supported the lipid titer of around 0.015 g/L by *R. opacus* PD630 or DSM 1069 [30].

*Rhodococcus jostii* RHA1 has one of the largest sequenced bacterial genomes of 9,702,737 bp encoding multiple biosynthesis pathways. Besides essential gene clusters for the metabolism and accumulation of polyhydroxyalkanoates (PHA), glycogen, and polyphosphate, *R. jostii* RHA1 also possesses key genes for TAG biosynthesis [31] with up to 55% CDW TAGs accumulated when fed on benzoate [22]. In addition, the whole genome sequence of *R. jostii* RHA1 suggests its versatile catabolism systems [32]. For catabolism of aromatic compounds, up to four aromatic central pathways and twenty-six predicted peripheral pathways [33] have been identified in *R. jostii* RHA1 showing its promising potential for the degradation of lignin-derived aromatics. Four aromatic central pathways include β-ketoadipate pathway encoded by the *pca* and *cat* genes, phenylacetic acid (PAA) pathway encoded by *paa* gene cluster, 2-hydroxypentadienoate (HPD) pathway encoded by *bphEFG* and *hsaEFG* genes, and an unidentified pathway comprised of a hydroxylase, an extradiol dioxygenase, and a hydrolase [32, 34–36].

**Fig. 1:**
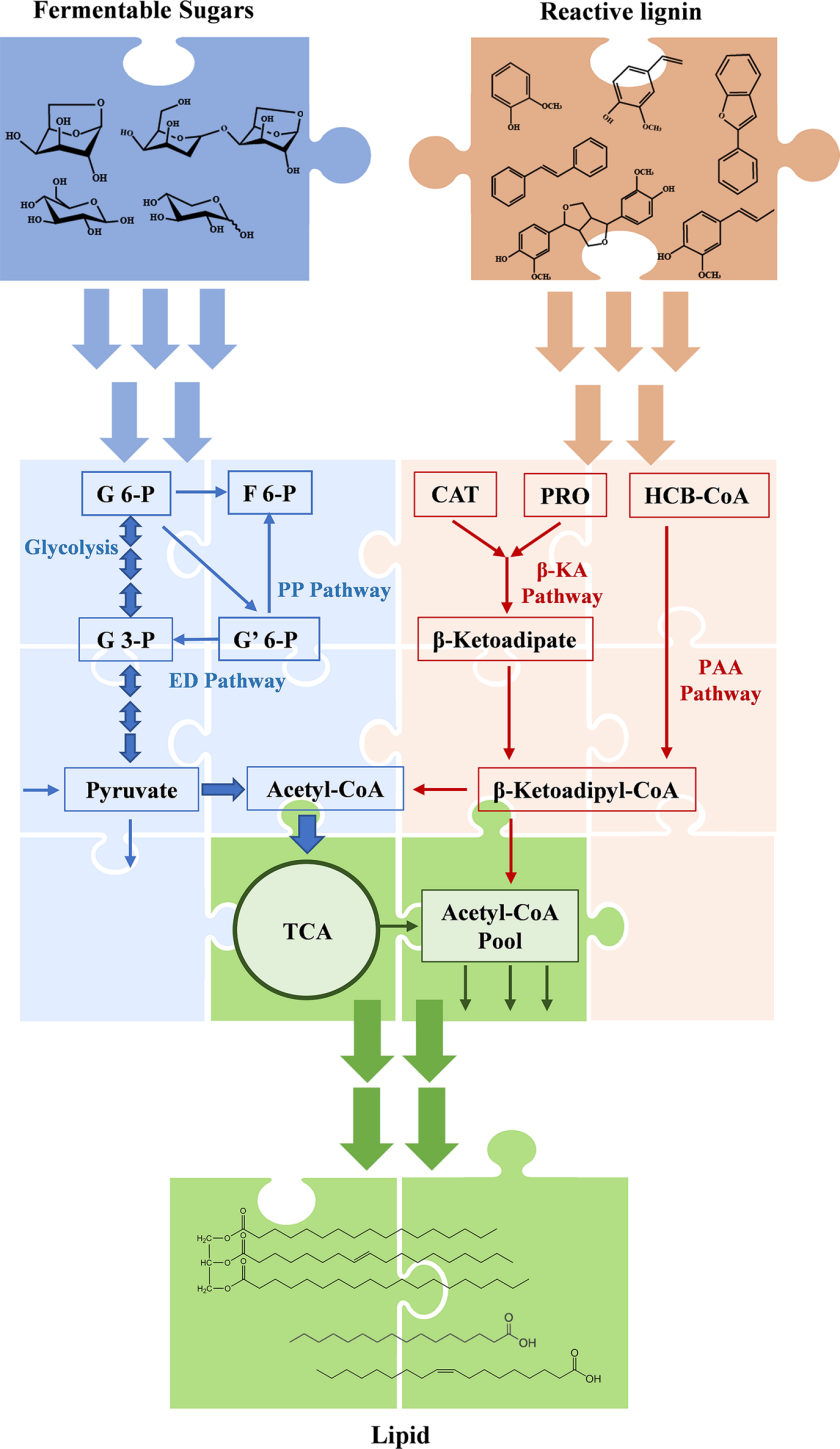
Integrated pathways in *Rhodococci* for conversion of both sugars and reactive lignin derived from dilute acid pretreated lignocellulosic biomass into lipid. G 6-P, Glucose 6-P; F 6-P, Fructose 6-P; G 3-P, Glyceraldehyde 3-P; G′ 6-P, Gluconate 6-P; CAT, catechol; PRO, protocatechuate; HCB-CoA, 2-Hydroxy-4-carboxy-butanyl-CoA; PP Pathway, Pentose Phosphate Pathway; ED Pathway, Entner–Doudoroff Pathway; β-KA Pathway, β-Ketoadipate Pathway; PAA Pathway, Phenylacetic acid Pathway

The β-ketoadipate pathway and PAA pathway represent two bacterial aerobic strategies to catabolize aromatic compounds, respectively: (1) employ various oxygenases to activate the benzene ring by catalyzing the substituted hydroxyl groups and yield central aromatic metabolites (e.g., catechol, protocatechuate), followed by ring cleavage between or adjunct to the hydroxyl groups; (2) utilize co-enzyme A (CoA) to reach the derivatization of aromatic acids without oxygenolytic ring fission (Fig. 1). The previous work demonstrated that higher degradation (~ 39.6%) of lignin was obtained by co-fermentation of *R. opacus* PD630 and *R. jostii* RHA1 VanA^−^ than that by single-strain fermentation [3]. It was suggested that the ferredoxin oxygenase (VanA) deletion mutant strain *R. jostii* RHA1 VanA^−^ [16] was able to metabolize lignin and the accumulated vanillic acid could be utilized as a carbon source by *R. opacus* PD630 via the β-ketoadipate pathway for lipid biosynthesis [37].

Significantly, biodegradation of lignin by co-fermentation with different lignin-degrading strains may enhance the depolymerization of lignin into aromatics and promote the lipid production. In view of different lignin degradation and/or lipid biosynthesis capacities, co-fermentation of different lignin-degrading strains may be used for the synergetic bioconversion of lignin into lipids. Grown together, these organisms (*R. opacus* PD630, *R. jostii* RHA1, and *R. jostii* RHA1 VanA^−^) have the potential to transform lignin into lipids efficiently.

Pretreatment plays a vital role in providing fermentable biomass compounds for biological processing of both carbohydrates and lignin to produce biofuels and value-added chemicals [4]. A flowthrough pretreatment of poplar wood at elevated temperatures (200–280 °C) revealed that hydrothermal pretreatment above 240 °C or with 0.05% (w/w) H_2_SO_4_ at 220 °C significantly disrupted and removed more than 98% total biomass [38], resulting in up to 100% of xylan and 90% of cellulose solubilized with negligible furfural and 5-HMF formation during the pretreatment. It was found that xylan was predominately removed as soluble xylo-oligomers with some xylose, and about 86% of glucan was removed as soluble glucose oligomers and glucose as well as the remaining glucan/cellulose readily for cellulases digestion with nearly 100% yield [38, 39]. Although about 98% of lignin was removed into liquid phase, most of them were re-precipitated when the pretreated hydrolysate was cooled down after the reactions [38]. Resinol, β-*O*-4′ linkage, and the phenylcoumaran structures with slight repolymerization with newly formed Cβ-C5′ linkages were observed in the precipitated lignin structure [40]. In addition, pretreatment was shown to mitigate lignin droplets onto the cellulose surface, and thus led to effective lignin deconstruction and hemicellulose recovery. The precipitated lignin from pretreated hydrolyzate had low molecular weight and was catalytically upgraded to C7–C18 range hydrocarbons through recent advances in hydrodeoxygenation [5, 41, 42]. Thus, through this advanced flowthrough pretreatment process, solubilized biomass slurries containing limited inhibitory compounds continuously exited the pretreatment system and were sequentially enzymatically hydrolyzed to carbohydrates (over 90% yield) along with low-molecular weight lignin as the unique carbon sources for biological processes to obtain biofuels and chemicals.

In this study, the selective combination of the wild-type and engineered *Rhodococcus* strains, including *R. opacus* PD630, *R. jostii* RHA1, and *R. jostii* RHA1 VanA^−^, with lignin degradation, aromatics utilization, and/or lipid biosynthesis capacities was used to co-ferment lignin and sugar (Fig. 1) to establish a fundamental understanding of the pathways and functional modules necessary to enable a platform for biological conversion of biomass-derived lignin into lipids. In addition, fermentation kinetics and proteomics analysis were carried out to identify potential catabolic pathways in co-fermentation for funneling biosynthesis of TAGs from both of lignin and carboxylates.

## Results

### Lignin degradation by single strain- or co-culture of *Rhodococci*

**Fig. 2:**
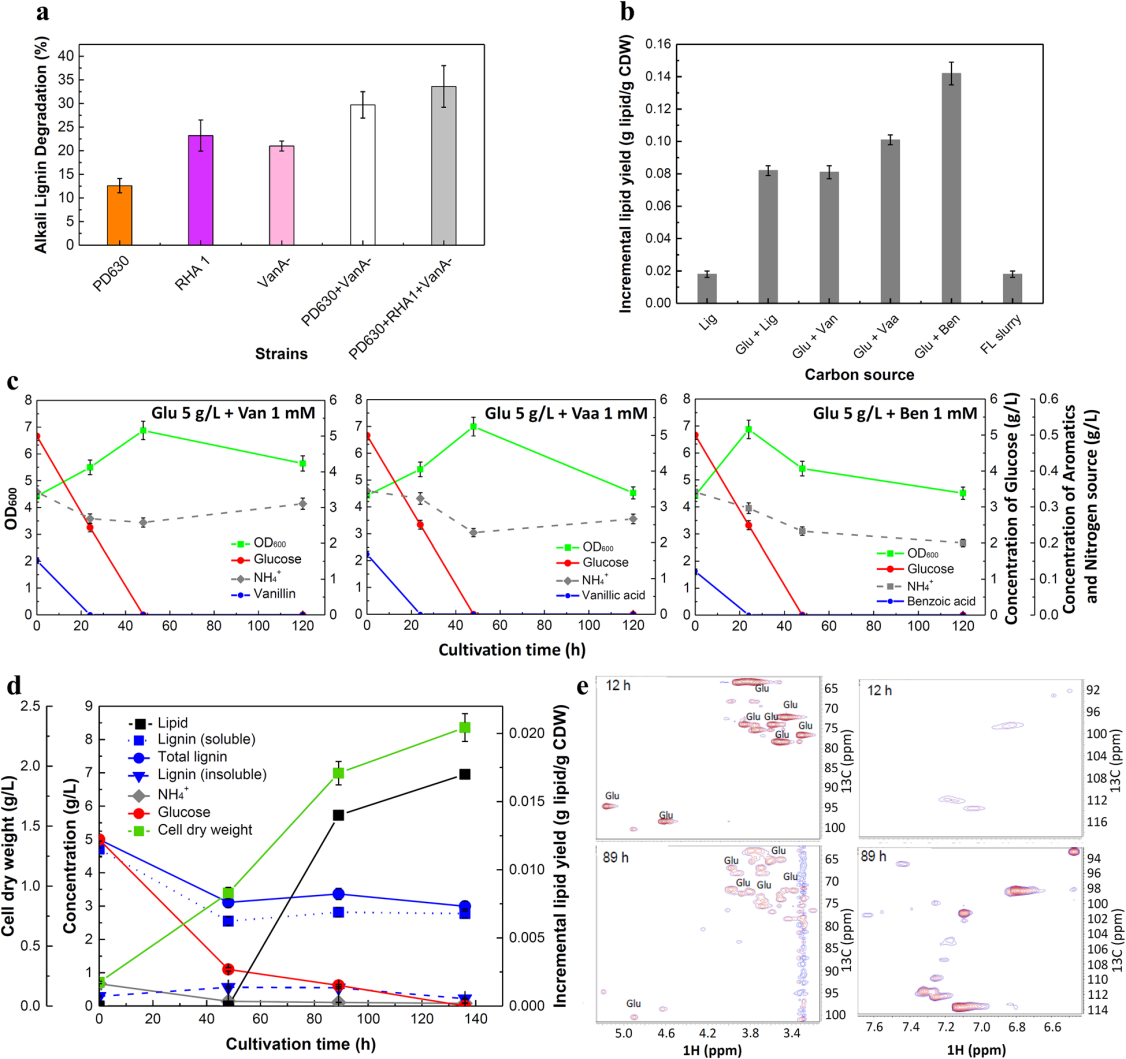
Biological conversion of carbohydrate and lignin (or lignin derivatives) into lipids by *Rhodococci*. **a** The comparison of lignin degradation by single- or co-culture of *R. opacus* PD630 (simplified as PD630 as presented), *R. jostii* RHA1 (simplified as RHA1), and/or *R. jostii* RHA1 VanA^−^ (simplified as VanA^−^). **b** Effects of carbon sources on the lipid content. The abbreviation of the names of carbon sources is used in the figure. The full names from left to right are: alkali lignin 5 g/L (control experiment); glucose 5 g/L + alkali lignin 5 g/L; glucose 5 g/L + vanillin 1 mM; glucose 5 g/L + vanillic acid 1 mM); FL slurry (glucose 5 g/L + pretreated lignin 0.593 g/L + alkali lignin 4.41 g/L). **c** Effects of carbon sources on the cell growth, glucose conversion and (NH_4_)_2_SO_4_ consumption. The left, glucose 5 g/L + vanillin 1 mM; the middle, glucose 5 g/L + vanillic acid 1 mM; the right, glucose 5 g/L + benzoic acid 1 mM. **d** Time courses for cell growth, carbohydrate conversion, lignin consumption, (NH_4_)_2_SO_4_ utilization and lipid production using carbohydrate and lignin from the enzymatic hydrolysis of flowthrough hydrolysates (FL slurry) as the carbon source. **e** ^1^H-^13^C HSQC-NMR analysis of fermentation supernatant from the co-culture of *R. opacus* PD630, *R. jostii* RHA1, and *R. jostii* RHA1 VanA^−^ on carbon source of FL slurry. The left two graphs show the chemical shift region of carbohydrates while the graphs on the right show the chemical shift region of aromatics

It was reported that the synergy of *Rhodococcus* strains during co-culture may promote lignin conversion into lipid [3]. In this study, lignin degradation by single-strain culture or co-culture of *R. jostii* RHA1, *R. jostii* RHA1 VanA^−^, or *R. opacus* PD630 was first examined to select the best combination of candidates for fermentation. The fermentation using *R. jostii* RHA1 individually achieved 23.2% of lignin degradation which was the best performance among single-strain cultures. Compared to the single-strain culture, or the two-strain co-culture of PD630 and VanA^−^ [3], the co-culture of three strains showed the best lignin degradation activity (33.6%) (Fig. 2a). Therefore, three-strain co-culture was used for further investigation.

### Conversion of mixed carbon sources of carbohydrate and lignin (or lignin model compounds) for lipid production

To investigate viability of the fermentation using the supplemented whole flowthrough-pretreated slurry followed by enzymatic hydrolysis (simplified as FL slurry in the following content) as substrate of carbohydrates and lignin by *Rhodococci*, 6-day fermentation of mixed carbon sources of glucose and lignin model compounds (including vanillin, vanillic acid, and benzoic acid), or alkali-pretreated corn stover lignin (simplified as alkali lignin in the following content) was examined as well. Various mixed carbon sources, including: (1) 5 g/L glucose + 5 g/L alkali lignin; (2) 5 g/L glucose + 1 mM vanillin; (3) 5 g/L glucose + 1 mM vanillic acid; (4) 5 g/L glucose + 1 mM benzoic acid; (5) FL slurry (glucose 5 g/L + 0.593 g/L pretreated lignin + alkali lignin 4.41 g/L, were used for lipid production by co-culture of *R. opacus* PD630, *R. jostii* RHA1, and *R. jostii* RHA1 VanA^−^. Five g/L of alkali lignin was used as the sole carbon source in the control experiment.

All tested carbon sources could be used for lipid accumulation under nitrogen-limiting condition (*C*/*N* = 15/1, g/g). Mixing glucose with benzoate promoted the highest incremental lipid production in cells (0.14 g lipid/g CDW). Glucose mixed with vanillic acid resulted in the second highest lipid yield of 0.10 g lipid/g CDW (Fig. 2b). However, using glucose mixed with vanillin had relatively less lipid production than that of other two lignin model compounds, and was comparable to the lipid production using alkali lignin in the presence of glucose, which may be due to the toxicity of aldehyde group of vanillin [37, 43]. It, thus, suggested that some lignin-derived monomers performed better for lipid production than the macro-molecules of pure lignin, and the structure of monomers may be a key to achieve high yields [1, 14]. Except for FL slurry as substrate, all mixed carbon sources gained higher incremental lipid yield compared to that using alkali lignin as the sole carbon source, which suggested that the presence of glucose promoted the lipid production in cells. Compared to using same amount of glucose mixed with lignin, a decrease of TAG accumulation during the co-fermentation of the whole FL slurry was observed. It can be caused by the appearance or high concentration of some by-products generated during pretreatment and hydrolysis process such as the carbohydrate-derived acids, and furans or lignin-derived aromatics [37, 44].

In the presence of each lignin model compound at 1 mM, 5 g/L glucose supported rapid cell growth and was completely consumed within 48 h (Fig. 2c). The highest optical density at 600 nm (OD_600_) reached around 7.0 at 48 h using vanillin or vanillic acid mixed with glucose as carbon sources. However, cells grew faster by mixing benzoic acid with glucose, which led to OD_600_ ~ 6.9 at 24 h. All these three model compounds (benzoic acid, vanillin, vanillic acid) were exhausted before 24 h. We further monitored the process of fermentation using FL slurry as the substrate (Fig. 2d). Glucose was utilized more quickly than lignin, which was consumed up around 136 h. Within the first 48-h fermentation, the amount of insoluble lignin in the medium dropped significantly, while the concentration of soluble lignin increased suggesting the modification of lignin structure. 40.1% of lignin was degraded in total by the strains at the end of fermentation. Lipid started to accumulate in the cells gradually when little (NH_4_)_2_SO_4_ was detected. At 136 h, the lipid content in cells was 0.0165 g lipid/g CDW which was comparably lower than that of using alkali lignin mixed with glucose as substrate, despite that same concentrations (5 g/L) of lignin or glucose were added in the fermentation medium (Fig. 2b). Furthermore, palmitic acid (C16:0) and oleic acid (C18:1) were the major FAMEs species in cells (Table 1) converted from FL slurry. The pH value during the fermentation was relatively stable, remaining at about 6.9–7.

**Table 1:** Distribution of accumulated fatty acids in cells during fermentation of FL slurry

Compounds	C14:0	C16:0	C16:1	C18:0	C18:1
Percent (wt%)-48 h	3.45 ± 0.17	51.72 ± 2.59	11.21 ± 0.56	12.07 ± 0.60	21.55 ± 1.08
Percent (wt%)-89 h	5.00 ± 0.25	41.87 ± 2.09	11.25 ± 0.56	17.50 ± 0.88	24.38 ± 1.22

Myristic acid (C14:0), palmitic acid (C16:0), palmitoleic acid (C16:1), stearic acid (C18:0), and oleic acid (C18:1) were determined by GC/MS

### Characterization of glucose and lignin metabolism during fermentation of FL slurry

To better understand the substrate utilization of complex component, fermentation supernatant was further characterized. ^1^H-^13^C HSQC-NMR analysis of the supernatant suggested that *Rhodococci* had preference to use glucose over lignin (Fig. 2e). In the early stage of fermentation (12 h), abundant glucose (top-left graph) while little aromatic lignin monomers (top-right graph) were found in the supernatant. At 89 h of the fermentation, most glucose was consumed (bottom-left graph), and aromatics derived from lignin showed stronger signals in the aromatic region (bottom-right graph). This result strongly supported that lignin was degraded during the fermentation.

The newly generated aromatic compounds include aromatic benzoic acid, which was also suggested by GC/MS result (Additional file 1: Figure S1). Due to the limitation of the GC/MS method, additional soluble lignin-derived compounds were not detected, especially oligomers. The broad peaks of aromatic compounds in Additional file 1: Figure S2 suggested that the soluble lignin intermediates in the fermentation supernatants were a mixture of monomers and oligomers which could not be individually identified yet. The comparison of the spectra between the samples at 89 h and 168 h post-inoculation also implied the generation and consumption of aromatics during the fermentation process.

### Proteomics analysis of enzyme systems for glucose or lignin metabolism by *Rhodococci*

Among previous omics studies that investigated the metabolism of carbohydrates or aromatics (lignin analogs) and TAG accumulation by *R. jostii* RHA1 or *R. opacus* PD630 [22, 27, 45], few proteomics studies of lignin metabolism was reported. To identify potential enzymes contributing to glucose or lignin utilization during co-culture, the global proteome profiles of cell lysates when strains were fed on glucose or lignin as the sole carbon source for 5 days were compared. To identify statistically significant changes, the Benjamini–Hochberg procedure was applied to control the false discovery rate (FDR) among multiple comparison [46, 47]. In total, 2740 proteins were identified. Compared with glucose fermentation, in the lysates of lignin one, there were 171 proteins up-regulated and 200 proteins down-regulated (fold-change ≥ 2, FDR < 0.1) (Additional file 2: Table S1).

**Fig. 3:**
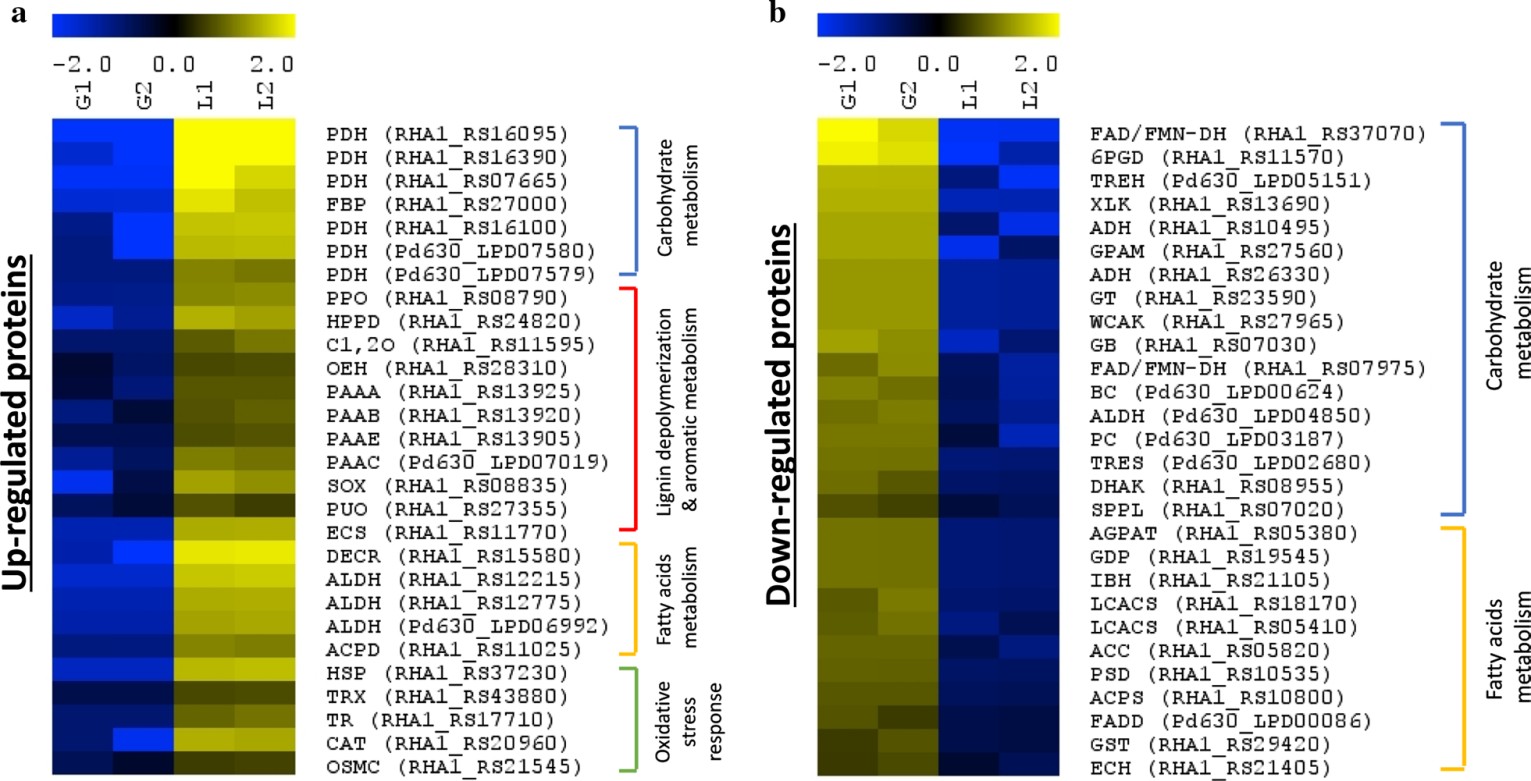
Overview of the differentially expressed proteins (FDR < 0.1, and fold-change ≥ 2.0) among the lysate samples from glucose or lignin fermentation after 5 days. Relative abundances were log2 transformed. Each row represents one protein and each column represented one sample. “G1” and “G2” are the lysate duplicate samples from glucose fermentation; “L1” and “L2” are the lysate duplicate samples from lignin fermentation. All the fermentation was conducted by co-culture of three strains: *R. jostii* RHA1, *R. jostii* RHA1 vanA^−^, *R. opacus* PD630. The gene names of strains follow the protein names abbreviation: gene names starting with “RHA1” = *R. jostii* RHA1 or *R. jostii* RHA1 vanA^−^ while gene name starting with “Pd630” = *R. opacus* PD630 (Additional file 3). **a** The selected up-regulated proteins. **b** The selected down-regulated proteins. A list of abbreviations can be found in Additional file 3

The up-regulation of 3-phenylpropionate/trans-cinnamate dioxygenase (PPO, RHA1_RS08790) and 4-hydroxyphenylpyruvate dioxygenase (HPPD, RHA1_RS24820) elucidated potential peripheral aromatic degradation pathways and possible lignin-derived monomers involved in lignin degradation by *Rhodococci* (Fig. 3a, Additional file 2: Table S1). Two central aromatic degradation pathways of *Rhodococci* were both activated during lignin fermentation, including phenylacetic acid (PAA) pathway [35] and β-ketoadipate pathway [34]. Several enzymes (PAAA, RHA1_RS13925, PAAB, RHA1_RS13920, PAAE, RHA1_RS13905, PAAC, Pd630_LPD07019) performing aromatic ring cleavage activity in the PAA pathway were up-regulated (Fig. 3a). The catechol 1,2-dioxygenase (C1, 2O, RHA1_RS11595) within the catechol branch of the β-ketoadipate pathway showed up-regulation of 2.8-folds, while no enzyme involved in the protocatechuate branch was significantly up-regulated (Fig. 3a). However, the fold-changes of enzymes involved in β-ketoadipate pathway were higher than those in PAA pathway, which indicated that the regulation of β-ketoadipate pathway might be more sensitive to lignin as the carbon source (Additional file 2: Table S1). Furthermore, 2-keto-4-pentenoate hydratase (OEH, RHA1_RS28310) were up-regulated 2.3-folds, suggesting the meta-cleavage of catechol may occur as well [48].

On the contrary, the main pathways related to glucose metabolism, including glycolysis, the pentose phosphate (PP) pathway, and the Entner–Doudoroff (ED) pathway, were greatly down-regulated when lignin was the sole carbon source, indicating that all these three pathways were plausibly involved in glucose utilization by *Rhodococci* (Fig. 3b), consistent with previous report [45]. In addition, some ATP-binding cassette (ABC) transporters of carbohydrates were less expressed (Additional file 2: Table S1; Additional file 5).

Several oxidases that can generate hydrogen peroxide to facilitate lignin depolymerization were differentially expressed [49, 50]. Sarcosine oxidase (SOX, RHA1_RS08835) was up-regulated 4.9-folds, and putrescine oxidase (PUO, RHA1_RS27355) was 2.2-fold up-regulated in *R. jostii* RHA1. Meanwhile, many proteins involved in oxidative stress response systems such as thioredoxin system and catalase were up-regulated during the lignin fermentation (Fig. 3a). The results, thus, suggested that the biodegradation of lignin involved oxidative conditions induced by hydrogen peroxide-producing enzymes, leading to the activation of the antioxidant mechanism in *Rhodococci*, which has also been observed in the fungal systems that produce lignin peroxidase (LiP) [51, 52].

The metabolism of fatty acids was also found more active during the fermentation of lignin than that of glucose, especially for fatty acid β-oxidation, which breaks down the fatty acids and generates NADH and acetyl-CoA as products for cell growth and substrate utilization [17]. In addition, enzymes involved in TAG biosynthesis were largely down-regulated during lignin fermentation compared with those during glucose fermentation such as long-chain acyl-CoA synthetase (RHA1_RS18170, RHA1_RS05410), and 1-acyl-sn-glycerol-3-phosphate acyltransferase (RHA1_RS29195) of Kennedy Pathway (Fig. 3b). It may be caused by the insufficient supply of reducing power and metabolites derived from lignin catabolism and degradation. Moreover, our results (Fig. 3a, Additional file 2: Table S1) are consistent with previous report that cellular oxidative stress decreased the abundance of key proteins of fatty acid biosynthesis and induced the up-regulation of proteins involved in β-oxidation [51]. Meanwhile, several components (RHA1_RS16095, RHA1_RS16390, RHA1_RS07665, RHA1_RS16100, PD630_LPD07580, PD630_LPD07579) of the pyruvate dehydrogenase complex (PDC)1 which link the glycolysis pathway to the TCA cycle were up-regulated. This is possibly regulated by the lack of ATP, NADH, or acetyl-CoA [33], which is consistent with the detection of fatty acid degradation activity in our results.

We further analyzed the extracellular secretome when strains were cultivated on glucose or lignin as the sole carbon source. In total, ~ 1800 proteins were identified. Due to the large differences in cell number during the glucose or lignin fermentation, the secretome data were considered as qualitative. However, many intracellular proteins which were up-regulated during lignin fermentation were also observed to be secreted, including several proteins involved in fatty acids metabolism, and aromatic degradation pathways (Additional file 2: Table S1). Nevertheless, the dye-decolorizing peroxidase B (DypB, RHA1_RS11765) of *R. jostii* RHA1 which was reported breaking the β-*O*-4 bonds of lignin model compound was identified in the secretome samples from lignin fermentation. The encapsulin protein (RHA1_RS11770) related to the DypB assembling which was up-regulated in vivo (Fig. 3a) was identified as well [53–55]. Many H_2_O_2_-generating oxidases besides sarcosine oxidase and putrescine oxidase were detected in the secretome (Additional file 4: Table S3), providing the essential mediator for the function of peroxidases, although further quantification of these enzymes is needed. Furthermore, glutathione peroxidase (RHA1_RS23555) and superoxide dismutase (RHA1_RS19475) of *R. jostii* RHA1 were identified, which may result from high concentration of peroxide or reactive oxygen species (Additional file 2: Table S1) [52] or involved in lignin oxidation [56]. Meanwhile, a catalase–peroxidase, which was reportedly involved in lignin degradation [49, 57] was secreted by both *R. jostii* RHA1 (RHA1_RS25800) and *R. opacus* PD630 (Pd630_LPD01797) (Additional file 2: Table S1).

## Discussion

Besides numerous studies on carbohydrate-based biofuel production, lignin valorization has attracted increasing attentions to obtain high carbon conversion of the entire process. Previous studies investigated various aromatic degradation pathways of possible lignin-derived monomers in wild-type or engineered strains to produce lipids, PHAs, or biochemicals from lignin model compounds or mixed biomass-derived lignin substrates [14, 16, 58–60]. However, the production capability from lignin of strains is not comparable with that of carbohydrates, and the knowledge on converting lignin into valuable products is limited. Herein, we studied the case of co-fermentation of lignin and carbohydrates derived from real lignocellulosic biomass by mixed wild-type and engineered *Rhodococci* for lipid production.

*Rhodococcus* strains are known for promising potential of bioremediation and lignin valorization due to their great catabolic capabilities of utilizing broad range of compounds [32]. Indeed, the tested lignin model compounds were rapidly utilized simultaneously with glucose, despite of the lower consumption rate. During the fermentation using dilute acid-pretreated biomass slurry supplemented with alkali lignin, the concentration of lignin decreased by almost half within 48 h (Fig. 2d), which is comparably more efficient than those of previous studies [3, 15, 24] in terms of higher depolymerization rate or shorter fermentation time. We proposed that mixed carbon source of glucose and lignin as well as co-culture of three *Rhodococcus* strains contributed to this result. The metabolism of glucose in *R. jostii* RHA1 and *R. opacus* PD630 goes through the glycolysis pathway, ED pathway, and PP pathway (Additional file 2: Table S1) [45, 61]. The supplementation of glucose to lignin may compensate the energy (in the form of ATP and NADH) for aromatic degradation pathways through the glycolysis pathway, while the ED pathway and the PP pathway generate NADPH for fatty acids synthesis [45, 62–64].

**Fig. 4:**
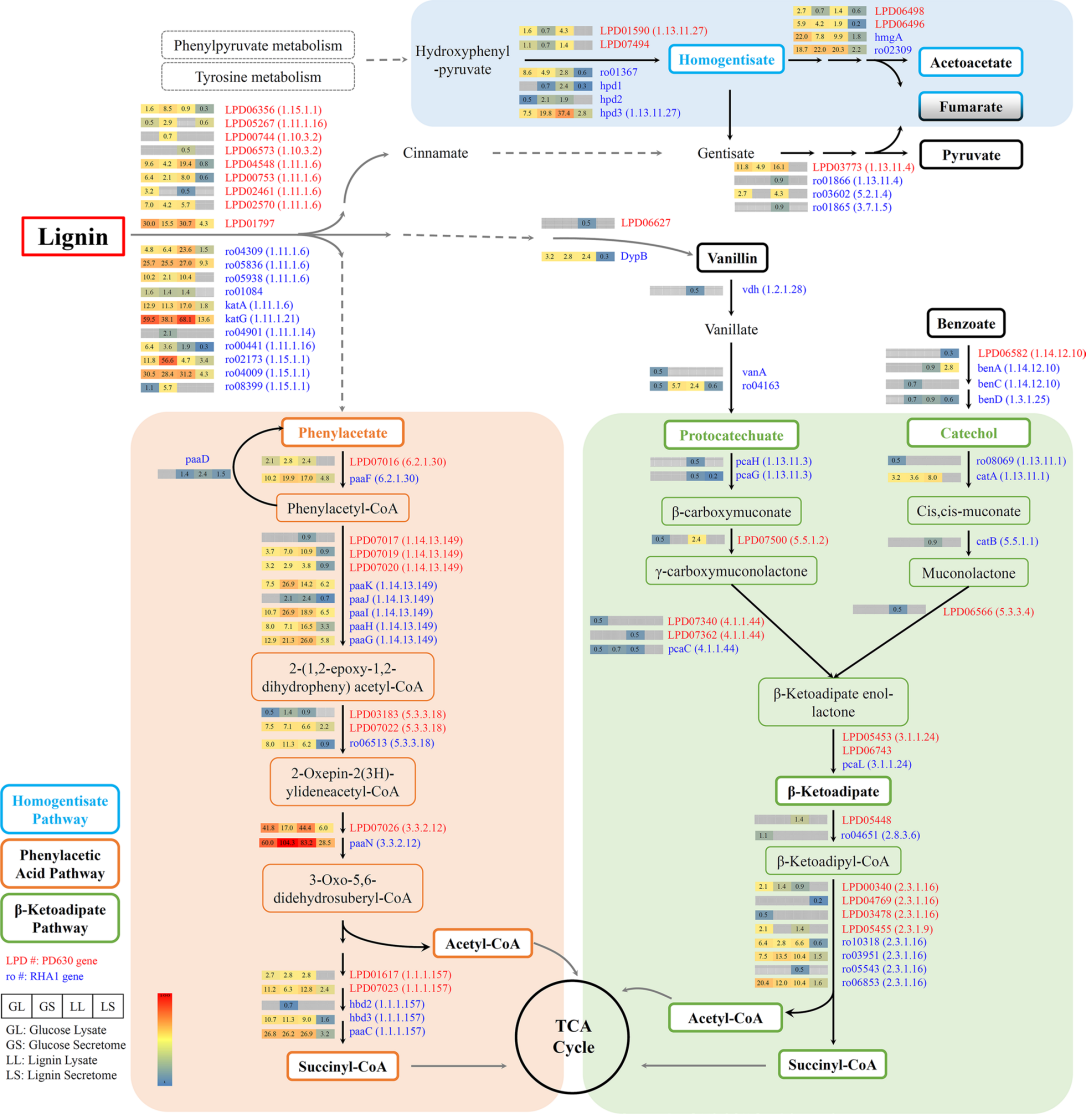
Proposed pathways of lignin degradation in *Rhodococci*. The genes followed by E.C. number of encoded enzymes of the key pathways were provided in red or blue color referring to the genes of *R. opacus* PD630 or *R. jostii* RHA1 (or *R. jostii* RHA1 vanA^−^), respectively. The numbers in the boxes, matched with the color of the boxes, were the level of protein abundance in four different samples: lysate samples of glucose fermentation (GL), secretome samples of glucose fermentation (GS), lysate samples of lignin fermentation (LL), and secretome samples of lignin fermentation (LS)

**Fig. 5:**
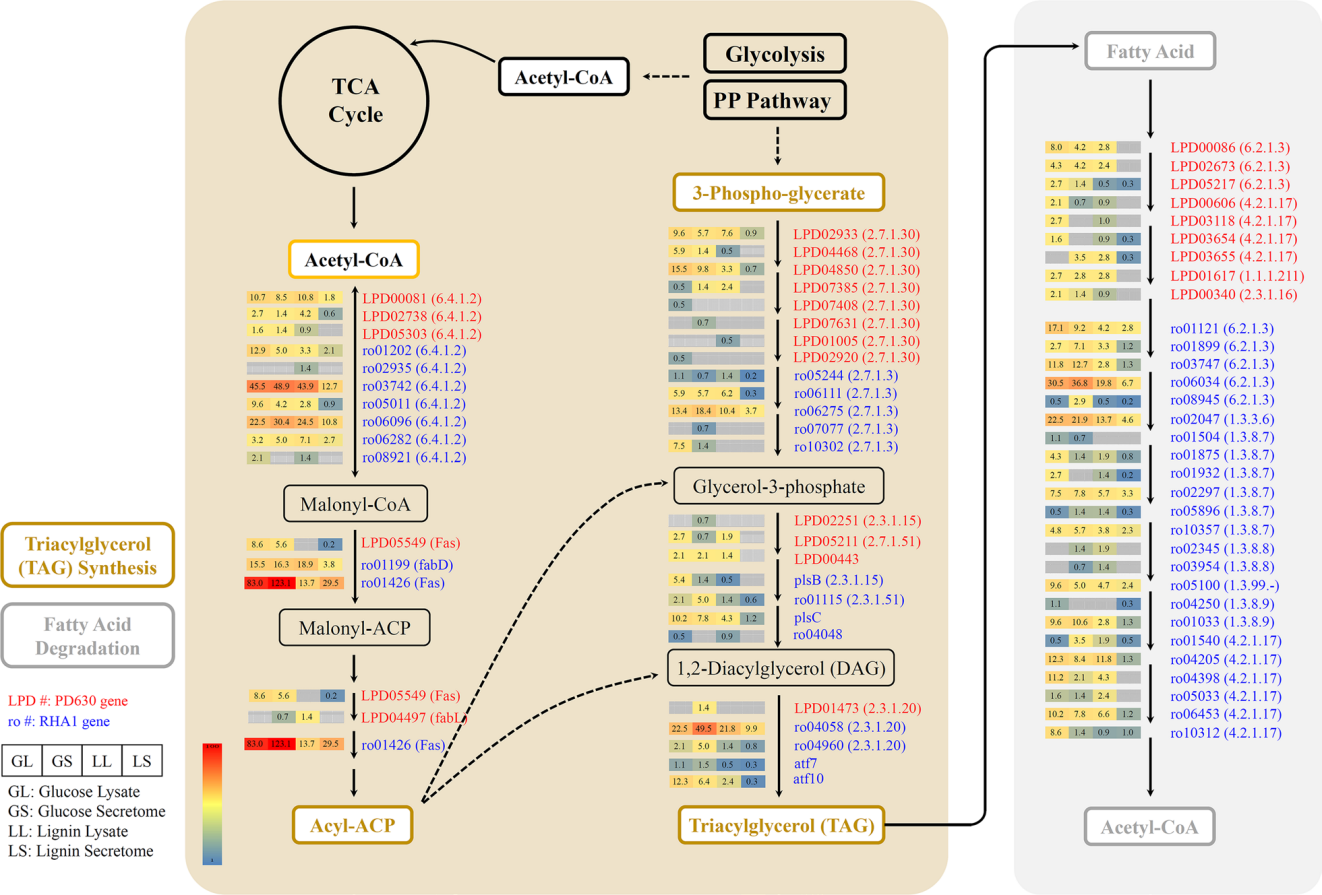
Proposed pathways of fatty acid metabolism in *Rhodococci.* The same layout was applied as in Fig. 4

Meanwhile, compared with single culture of each strain or co-culture of *R. opacus* PD630 and *R. jostii* RHA1 vanA^−^, the co-culture of three strains in this study showed the highest activity of lignin conversion (Fig. 2a), which may be due to the synergy among *R. opacus* PD630, *R. jostii* RHA1, and *R. jostii* RHA1 vanA^−^. Although the in vivo aromatic degradation pathways of all three strains were similar (Figs. 4, 5, Additional file 1: Figures S4, S5), the extracellular depolymerization of lignin was different. Despite the non-comparable cell density, the catalase–peroxidase of *R. jostii* RHA1 was up-regulated around twofold which was higher than the fold-change of that of *R. opacus* PD630 (Additional file 2: Table S1). Also, many of the possible lignin-degrading related enzymes we detected were originated from *R. jostii* (Additional file 3: Tables S1, S2). These observations may suggest that during co-fermentation, *R. opacus* PD630 had lower extracellular activity during lignin fermentation compared with *R. jostii* RHA1 and *R. jostii* RHA1 vanA^−^. Thus, the co-culture of *R. opacus* PD630 with other two strains may help *R. opacus* PD630 get access to lignin-derived products as carbon sources for cell growth and lipid production as discussed in our previous work [3]. However, more detailed work such as secretomic or genetics study is needed to confirm the synergic effect of co-culturing these strains.

To our knowledge, the peroxidase DypB is the only characterized lignin-degrading enzyme in *R. jostii* RHA1. An encapsulin protein was reported to be encoded by a gene located immediately downstream of *dypB* gene and packed DypB by binding its terminal targeting peptide. However, the location of DypB–encapsulin complex remains unresolved. The encapsulin nanocompartment has been observed only intracellularly in bacteria with some exceptions: the encapsulin-related linocins from *B. linens* and *M. tuberculosis* were detected extracellularly [53–55]. Our observation suggested the secretion of DypB–encapsulin complex by *R. jostii* RHA1, which needs further characterization.

In previous studies, *R. jostii* RHA1 showed consistent activity of lignin degradation in the absence of hydrogen peroxide, suggesting that *R. jostii* RHA1 may possess the ability of H_2_O_2_ generation similar to the fungal system to assist the activity of peroxidases or extracellular ligninases like laccases which uses O_2_ for oxidation [13, 65–67]. Herein, we demonstrated that sarcosine oxidase and the putrescine oxidase were up-regulated in vivo during lignin co-culture fermentation. However, the diffusion of H_2_O_2_ across cell membrane by bacteria can be limited [68, 69]. Our secretome results confirmed that they were secreted by *Rhodococci* as accessory enzymes for peroxidase activity. Besides these two oxidases, many other oxidases were identified in the secretome as well for H_2_O_2_ generation (Additional file 4: Table S3). The identification of glutathione peroxidase, catalase–peroxidase, and superoxide dismutase which act as the first line of defense also proved the in vitro presence of H_2_O_2_ or other reactive oxygen species resulting in oxidative stress [51, 70], while further quantification and characterization are needed to determine their involvement in extracellular lignin depolymerization. Notably, both catalase–peroxidase and superoxide dismutase were reported to modify phenolic lignin model compound or organosolv and kraft lignin, respectively [49, 56, 57], which suggested the potential synergy of various enzymes for lignin oxidation activity. On the other hand, H_2_O_2_ and reactive oxidative/aromatic radicals (e.g., hydroxyl radical, veratryl alcohol cation radical) were proposed as strong oxidizers to initiate the attack on lignin through non-enzymatic reactions [65, 71, 72]. Further investigation is needed on the correlation among concentration of H_2_O_2_ and reactive oxygen species and lignin depolymerization.

Overall, our results suggested that *Rhodococci* mobilized a multi-peroxidases system for lignin depolymerization with the assistance of oxidases under strong oxidative condition. This also implied a synergistic system of both enzymatic and chemical reactions, which shared common with *Pseudomonas putida* A514 possessing a dye peroxidase-based enzymatic system along with a redox-cycling reaction for hydroxyl radical generation proposed by Lin et al. [73]. Nevertheless, the existence of other types of ligninases cannot be denied. Low laccase activity of *R. jostii* RHA1 was detected by Salvachúa et al. [24]. Herein, we identified a multicopper oxidase with three cupredoxin domains in the secretome sample (Additional file 4: Table S2), which was predicted to be a bacterial endospore coat component CotA belonging to the laccase-like multicopper oxidase family based on the conserved domain hits within NCBI database. It may share similarity with the best-studied bacterial laccase CotA of *Bacillus subtilis* participating in the pigmentation of spores and providing protection of spore from UV light and H_2_O_2_ [74], which need further detailed identification and characterization.

Lignin-derived aromatic single-ring compounds such as vanillin, *p*-coumaric acid, ferulic acid, 4-hydroxybenzoate, etc. which yielded protocatechuate and entered the β-ketoadipate pathway were proposed and discussed in previous studies [3, 58, 64]. In our work, the catechol branch of the β-ketoadipate pathway was identified, suggesting other possible intermediates produced from lignin. It may be benzoate or its analog as the benzoate transporter (RHA1_RS14235) was up-regulated 4.5-fold (*p* = 0.028, FDR = 0.114) [75], which was identified by GC–MS as well (Additional file 1: Figure S1). Besides, 3-phenylpropionate, trans-cinnamate or their analogs can be lignin degradation products as well, and degraded by catalyzing the substituted hydroxyl groups in alternative peripheral pathway [76] followed by extradiol cleavage, which was in a good agreement with the up-regulation of 2-keto-4-pentenoate hydratase expressed by a *mph*D gene. Though the PAA pathway was not functionally characterized [33], it should play an important role in the in vivo catabolism of lignin-derived aromatics as a central aromatic degradation pathway in *Rhodococci* (Figs. 3a, 4) [35].

Furthermore, it was predicted that *Rhodococci* possessed the homogentisate pathway as characterized in *P. putida* [33, 77], and HmgB encoding a fumarylacetoacetate hydrolase within this pathway was up-regulated 1.4-fold during lignin fermentation. Despite it did not pass our test as a significant changed one, it still suggested another possible involved central aromatic degradation pathway since the central intermediate homogentisate can be provided by the activity of 4-hydroxyphenylpyruvate dioxygenase, which was up-regulated greatly in our results (Fig. 3a). Generally, it suggested a sophisticated and multichannel aromatics catabolism network to support the conversion of complex and heterogenous components derived from lignin, which further implied that the bacterial extracellular lignin-degrading activity may not be efficient enough to provide available and adequate aromatics taken in by cells to compete with glucose utilization.

Nevertheless, glucose was apparently the preferred carbon source of *Rhodococci*, which was consumed much faster than lignin (Fig. 2d). Also, the presence of glucose resulted in higher TAG accumulation during the co-fermentation than that using lignin as the sole carbon source. *R. opacus* PD630 was reportedly significant capable of lipid production from glucose [78]. However, using lignin or lignin model compounds, less TAG was obtained by *Rhodococci*; the titer was small as well [1, 14]. To promote the efficiency of lignin conversion into valuable products, many attempts have been made, such as modification of lignin structure before fermentation, genetical engineering of promising strains, novel fungal–bacterial or enzymatic–bacterial systems, etc. [1, 15, 21, 73]. In this study, the low lipid production from lignin may result from insufficient supply of reducing power and energy due to ineffective bacterial lignin catabolism system, and severe competing for NADPH between TAG accumulation and oxidative stress response. We demonstrated that the co-culture of *Rhodococcus* strains and co-fermentation of lignin with nutrient-rich substrates like glucose can be a new strategy for lignin valorization. Considering the cost of glucose is high, using lignocelluloses-pretreated hydrolysates is an alternative substrate containing both carbohydrates and lignin to produce lipid or other value-added commodity products. However, it requires more detailed studies to characterize the effect of its complex components on cell growth and carbon flux capacity to target products, followed by optimization of cultures and conditions to make a viable process.

## Conclusions

The development of effective lignin valorization is a key solution to improve the carbon efficiency of the entire process of biorefinery. In this study, the co-fermentation of lignin and carbohydrates by co-culture of wild-type and engineered *Rhodococci* for lipid production was investigated. Carbon utilization preference of glucose over lignin was observed. Lignin model compounds (vanillin, vanillic acid, and benzoic acid) were rapidly consumed as well, suggesting the bacterial lignin conversion may be limited by the comparably weak extracellular depolymerization activity to produce reactive lignin molecules as viable carbon sources for cell growth or energy storage. The low lipid production and up-regulation of β-oxidation of fatty acid degradation using lignin as the sole carbon source also showed the sign of lack of reducing power and energy. Nevertheless, 40.1% low-molecular weight lignin derived from dilute acid pretreated poplar wood was degraded, which could be supported by the extracellular peroxidases of *Rhodococci* with assistance of oxidases under strong oxidative conditions. However, the oxidative environment generated can be an alternative explanation of fatty acid degradation due to the competition for NADPH pool caused by the need of oxidative stress response system. It also suggested a combination of enzymatic and chemical reactions of lignin depolymerization. A strong in vivo aromatic degradation network provided a variety of paths, including both the β-ketoadipate pathway and the phenylacetic acid pathway to overcome the heterogeneity of lignin-derived aromatics. Collaboration of strains and synergistic pathways of sugar and aromatic metabolism were proposed, suggesting a promising basis for the design of the synergistic platform for the lignocellulosic biofuel production.

## Methods

### Dilute acid pretreatment of poplar wood

Poplar wood from Forest Concepts (Auburn, WA, USA) contains 48.8% cellulose, 16.8% xylan, and 23.7% Klason lignin as determined following the standard National Renewable Energy Laboratory Analytical Procedures (NREL LAPs) [79]. Poplar wood material was grounded by Hammer1067-A-1 (Hammermill, Buffalo, NY, USA) at 4500 rpm with a 1.59-mm screen, followed by sieving between 20 mesh and 40 mesh to obtain 0.425–0.850-mm biomass particles. 0.5 g of biomass was loaded into the tubular reactor which was attached to the flowthrough system (Additional file 4). The flowthrough system was set up as previously reported [38, 80]. 0.05% (w/w) sulfuric acid was pumped through the reactor at 240 °C for 8 min. The flow rate was set to 25 mL/min. Pretreated solids were subjected to enzymatic hydrolysis at pH 4.8, 50 °C for 24 h by Novozymes Cellic^®^ CTec2 (220 mg protein/mL, 205 FPU/mL) and Cellic^®^ HTec2 (230 mg protein/mL) as previously described [80]. 10-mg protein of Ctec2 (9.3 FPU) with 2-mg protein of Htec2 was used for per gram of glucan plus xylan. The resulted slurry was defined as flowthrough slurry, containing 1.27 g/L glucose, 0.47 g/L xylose, 0.013 g/L soluble lignin, and 0.58 g/L insoluble lignin. It was supplemented with 3.73 g/L glucose and 4.41 g/L alkali lignin to reach 5 g/L of each substrate and used for conversion into lipid by *Rhodococci* in this study. Insoluble lignin in the pretreated hydrolysates possessed the number average molecular weight (Mn) and the weight average molecular weight (Mw) of 1083 and 1955 Da, respectively [80].

### Alkali lignin preparation

Corn stover (50 kg) provided by the Idaho National Laboratory was extracted by 550 L of 0.1 M NaOH at 80 °C for 2 h to obtain lignin-rich solids, which consisted of 20% glucose, 11% xylose, 3% arabinose, 2% galactose, 53% lignin, and 11% ash. The lignin-rich solids were further soaked in 0.1 M NaOH solution (pH 12.5) to solubilize lignin, followed by filtration with a 11-μm-pore-size Whatman filter. Lignin was recovered by adjusting the filtrate to pH 3 with 2 M H_2_SO_4_. The precipitated lignin was collected by vacuum filtration and washed twice with deionized water at 70 °C, and then freeze-dried for 3 days.

### *Rhodococci* cultivation

The seed culture was inoculated with a single colony of *Rhodococcus* strains (*R. opacus* PD630, *R. jostii* RHA1 and its mutant *R. jostii* RHA1 VanA^−^) in Tryptic Soy Broth (TSB) medium and cultivated at 30 °C to OD_600_ ~ 1.5. The cell pellets were collected, washed twice with 0.9% (w/v) NaCl. Then, cells of each strain were inoculated in 100 mL RM medium at 5% (v/v, initial OD_600_ ~ 0.225) in 250 ml shaking flasks for fermentation at 30 °C, 180 rpm for 6 days. The RM medium (per liter) contains: MgSO_4_·7H_2_O 1.0 g, CaCl_2_·2H_2_O 0.015 g, 1.0 mL of sterile trace element solution, 1.0 mL of sterile stock A solution, and 35.2 mL of sterile 1.0 M phosphate buffer at pH 7.0. The trace element solution contains (per liter): CoCl_2_·6H_2_O 0.050 g, CuCl_2_·2H_2_O 0.0050 g, EDTA 0.25 g, FeSO_4_·7H_2_O 0.50 g, H_3_BO_3_ 0.015 g, MnSO_4_·H_2_O 0.020 g, NiCl_2_·6H_2_O 0.010 g, and ZnSO_4_·7H_2_O 0.40 g. Stock A solution contains 5.0 g/L FeNa-EDTA and 2.0 g/L NaMoO_4_·H_2_O [15]. The concentration of (NH_4_)_2_SO_4_ was modified for lipid production based on *C*/*N* = 15/1 (g/g, calculated by the concentration of carbon sources divided by 15). Five combinations of mixed carbon sources were added in the RM medium: (1) glucose (5 g/L) + alkali lignin (5 g/L); (2) glucose (5 g/L) + vanillin (1 mM, 0.152 g/L); (3) glucose (5 g/L) + vanillic acid (1 mM, 0.168 g/L); (4) glucose (5 g/L) + benzoic acid (1 mM, 0.122 g/L); (5) supplemented flowthrough-pretreated hydrolysates (FL slurry). Vanillin and other lignin model compounds were added to the final concentration of 1 mM, which is below the reported inhibition threshold of vanillin on the cell activity of *R. jostii* and *R. opacus,* to obtain robust growth of these strains during fermentation.

To monitor the microbial growth during fermentation, cell density at 600 nm (OD_600_) was analyzed with a UV/vis spectrophotometer (UV-2550PC, Shimadzu, Japan). The cell pellet was washed twice with 0.9% (w/v) NaCl, and then lyophilized in a VirTis lyophilizer (the VirTis Co., Inc., Gardiner, NY) to measure cell dry weight (CDW). Glucose in the hydrolyzates of pretreatment and enzymatic hydrolysis, as well as in fermentation broth, was analyzed by HPLC [81] (Additional file 5). The determination of ammonium concentration was conducted using phenol-hypochlorite method as previous reported [82]. The metabolites during fermentation were extracted by ethyl acetate and analyzed by GC–MS (Additional file 5). The pH change was monitored by a pH meter.

### Lignin characterization and quantification

2D ^1^H-^13^C heteronuclear single quantum coherence (HSQC)-NMR was acquired to characterize lignin during fermentation. 50.0-mg lignin sample was dissolved in 450-µL DMSO-d6 with 1-mg/mL Chromium (III) acetylacetonate as the relax reagent. A standard Bruker pulse sequence “hsqcetgpsi.2” was employed with a 90° pulse, 0.11-s acquisition time, a 1.5-s pulse delay, a 1JC–H of 145 Hz, 48 scans and acquisition of 1024 data points (for ^1^H) and 256 increments (for ^13^C). The ^1^H and ^13^C pulse widths were *p*1 = 11.30 µs and *p*3 = 10.00 µs, respectively. The ^1^H and ^13^C spectral widths were 13.02 ppm and 220.00 ppm, respectively. The central solvent peak was used for chemical shift calibration. HSQC-NMR data processing and plots were carried out using MestReNova v7.1.0 software’s default processing template and automatic phase and baseline correction.

One-dimensional ^13^C or ^1^H spectra were conducted. Quantitative ^13^C NMR was acquired using 50.0-mg lignin samples dissolved in 450-µL DMSO-d6 with 1-mg/mL Chromium (III) acetylacetonate. An inverse-gated decoupling pulse sequence (zgig), 90° pulse angle, a pulse delay of 12 s, and 5000 scans were employed for all the lignin samples. These samples were also analyzed using DEPT-135 ^13^C-NMR and DEPT-90 ^13^C-NMR. DEPT-135 ^13^C-NMR employed a standard Bruker pulse sequence “dept135” with a 135° pulse angle, 2-s pulse delay, and 5000 scans. DEPT-90 ^13^C-NMR employed a standard Bruker pulse sequence “dept90” with a 90° pulse angle, 2-s pulse delay, and 5000 scans. Also, Quantitative ^1^H-NMR was acquired for the lignin samples with 64 transients and 8-s pulse delay by employing a standard Bruker pulse sequence “zg” at room temperature.

The concentration of soluble or insoluble lignin in the fermentation broth was determined using the Prussian blue assay (Additional file 5).

### Lipid extraction and FAMEs analysis

To quantify the total lipid in cells, 50 mL of fermentation broth was centrifuged to collect the cells, which were then washed with 0.9% (w/v) NaCl and lyophilized to determine cell dry weight. 3 mL of chloroform and methanol mixture (chloroform: methanol = 2:1, v/v) was added to cells in a sealed testing tube and incubated at 30 °C, 180 rpm for 3 h, followed by adding 0.5 mL of deionized water. After phase separation, the bottom phase containing lipids was extracted again with 2 mL of chloroform and methanol mixture (chloroform: methanol: water = 3:48:47, v/v/v). The lower phase was collected and evaporated under N_2_ atmosphere and the weight of lipids was determined [15]. The weight of lipids was divided by cell dry weight to obtain the total lipid yield (g lipid/g CDW). Then, the incremental lipid yield (g lipid/g CDW) of cells was calculated by deducting the initial lipid yield (at 0 h fermentation) from the total lipid yield. The fatty acid methyl esters (FAMEs) were obtained by the sulfuric acid–methanol method [1]. The FAMEs distribution was determined using an Agilent 7890 GC coupled with Agilent 5975 mass spectrometer as previously reported [6] (Additional file 5).

### LC–MS/MS label-free proteomics analysis

5 g/L of glucose or lignin was used as sole carbon sources for fermentation of three *Rhodococcus* strains. After 5-day fermentation, cell pellets and supernatant were separated by centrifugation at 8000 rpm, 4 °C for 15 min. Cell pellets were then washed twice with 0.9% (w/v) NaCl solution and resuspended in lysis buffer (8 M urea, 75 mM NaCl in 100 mM NH_4_HCO_3_, pH 7.8), followed by 8 rounds of 30-s bead beating using a Bullet Blender (Homogenizers, Atkinson, NH) [83]. Lysate was collected and centrifuged at 10,000 rpm, 4 °C for 10 min to remove cellular debris and stored at − 80 °C for further digestion. The supernatant was concentrated using 30 KD filter (EMD Millipore, Billerica, MA) by centrifugation at 4000 rpm, 4 °C for 30 min. The concentrated supernatant was transferred to a clean tube and stored at − 80 °C for further digestion. The protein purification and digestion of all samples were conducted with the FASP Protein Digestion Kit (Expedeon, San Diego, CA) with trypsin (Promega, Madison, WI) following the manufacturer’s instruction. All samples were cleaned up by C18 SPE Clean-up column (Agilent technologies, Santa Clara, CA) and concentrated again by a Speed Vac SC110 (ThermoSavant, Holbrook, NY) following the manufacturer’s instructions. The protein concentration was estimated by the Pierce™ BCA protein assay (Thermo Scientific, San Jose, CA) and normalized to 0.1 µg/µL before LC–MS/MS analysis. Biological duplicates were applied during the entire process.

LC–MS/MS analysis was performed using a Q-Exactive HF Mass Spectrometer (Thermo Scientific, San Jose, CA). Data were acquired for 100 min when the gradient started. The peptide tandem mass spec raw data were searched against the FASTA files of all strains (*R. opacus* PD630, Accession: PRJNA30413; *R. jostii* RHA1, Accession: PRJNA309609) from NCBI and JGI database using MSGF+ algorithm for peptide identification [84]. The parent ion tolerance was ± 20 ppm. Partially tryptic termini were used for searching. The spectral level FDR was ≤ 1% based on a decoy database searching strategy [85].

Protein abundances were based on the spectral count data (i.e., the number of total observations for a given protein) and global central tendency normalization was applied across conditions [86]. Student *t*-test was applied to compare the protein expression differences [45]. The Benjamini–Hochberg procedure was applied to control the FDR for multiple testing [46, 47]. To identify statistically significant differences of protein expression between glucose and lignin as the sole carbon source, the following criteria were applied: (1) fold-change ≥ 2; (2) FDR < 0.1.

## Additional files

**Additional file 1: Figure S1.** GC/MS analysis of fermentation supernatant from the co-culture of *R. opacus* PD630, *R. jostii* RHA1, and *R. jostii* RHA1 VanA^−^ on carbon source of supplemented flowthrough-pretreated poplar whole slurry (glucose 5 g/L + pretreated lignin 0.593 g/L + alkali lignin 4.41 g/L). The chemical detected were as following: (1) 2,3-butanediol; (2) acetic acid; (3) acetaldehyde, hydroxy-; (4) methylglyoxal; (5) Butanoic acid, 3-hydroxy-, methyl; (6) 2-propanone, 1,3-dihydroxy-; (7) Benzoic acid; (8) Hexanoic acid, 3-hydroxy-, methyl; (9) 3-hydroxy-4-methyl-hexanoic acid. **Figure S2.** ^1^H-NMR analysis of fermentation supernatant from co-fermentation of *R. opacus* PD630, *R. jostii* RHA1, and VanA^−^ with carbon source of supplemented flowthrough-pretreated poplar whole slurry (glucose 5 g/L + pretreated lignin 0.593 g/L + alkali lignin 4.41 g/L) after 168 h (a), and 89 h (b). **Figure S3.** Gradient Selected 2D HSQC Analysis of Alkali lignin. **Figure S4.** Proposed detailed pathways of lignin degradation in *Rhodococci*. **Figure S5.** Proposed detailed pathways of fatty acid metabolism in *Rhodococci*.

**Additional file 2: Table S1.** Normalized global proteome data of spectral count for the lysate and secretome samples from glucose or lignin fermentation after 5 days. Each row represents one protein and each column represented one sample. “G1” and “G2” are the duplicate samples from glucose fermentation; “L1” and “L2” are the duplicate samples from lignin fermentation. All the fermentation was conducted by co-culture of three strains: *R. jostii* RHA1, *R. jostii* RHA1 vanA^−^, *R. opacus* PD630.

**Additional file 3.** A list of abbreviations is included.

**Additional file 4: Table S2.** Possible lignin-degrading related enzymes in the secretome of *Rhodococci* during lignin fermentation. **Table S3.** Oxidases in the secretome of *Rhodococci* during lignin fermentation.

**Additional file 5.** Supplemental information of methods is included.
