# Supplementary material for: Discovery of potential pathways for biological conversion of poplar wood into lipids by co-fermentation of Rhodococci strains
Source: Biotechnol Biofuels. 2019 Mar 19;12:60. doi: 10.1186/s13068-019-1395-x (PMC6423811; doi:10.1186/s13068-019-1395-x)
Supplement: Supplementary file 1 — Additional file 1: Figure S1. GC/MS analysis of fermentation supernatant from the co-culture of R. opacus PD630, R. jostii RHA1, and R. jostii RHA1 VanA− on carbon source of supplemented flowthrough-pretreated poplar whole slurry (glucose 5 g/L + pretreated lignin 0.593 g/L + alkali lignin 4.41 g/L). The chemical detected were as following: (1) 2,3-butanediol; (2) acetic acid; (3) acetaldehyde, hydroxy-; (4) methylglyoxal; (5) Butanoic acid, 3-hydroxy-, methyl; (6) 2-propanone, 1,3-dihydroxy-; (7) Benzoic acid; (8) Hexanoic acid, 3-hydroxy-, methyl; (9) 3-hydroxy-4-methyl-hexanoic acid. Figure S2. 1H-NMR analysis of fermentation supernatant from co-fermentation of R. opacus PD630, R. jostii RHA1, and VanA− with carbon source of supplemented flowthrough-pretreated poplar whole slurry (glucose 5 g/L + pretreated lignin 0.593 g/L + alkali lignin 4.41 g/L) after 168 h (a), and 89 h (b). Figure S3. Gradient Selected 2D HSQC Analysis of Alkali lignin. Figure S4. Proposed detailed pathways of lignin degradation in Rhodococci. Figure S5. Proposed detailed pathways of fatty acid metabolism in Rhodococci. [file 13068_2019_1395_MOESM1_ESM.docx]

Figure S1. GC/MS analysis of fermentation supernatant from the co-culture of *R. opacus* PD630, *R. jostii* RHA1, and *R. jostii* RHA1 VanA^-^ on carbon source of supplemented flowthrough pretreated poplar whole slurry (glucose 5 g/L + pretreated lignin 0.593 g/L + alkali lignin 4.41 g/L). The chemical detected were as following: 1) 2,3-butanediol; 2) acetic acid; 3) acetaldehyde, hydroxy-; 4) methylglyoxal; 5) Butanoic acid, 3-hydroxy-, methyl; 6) 2-propanone, 1,3-dihydroxy-; 7) Benzoic acid; 8) Hexanoic acid, 3-hydroxy-, methyl; 9) 3-hydroxy-4-methyl-hexanoic acid.


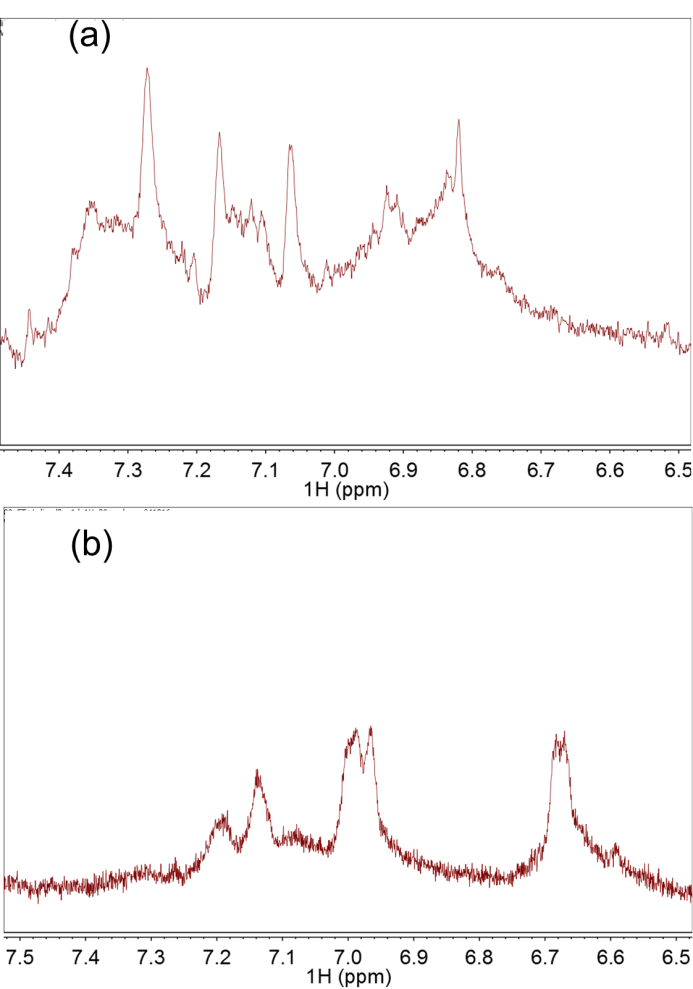


Figure S2. ^1^H-NMR analysis of fermentation supernatant from co-fermentation of *R. opacus* PD630, *R. jostii* RHA1, and VanA^-^ with carbon source of supplemented flowthrough pretreated poplar whole slurry (glucose 5 g/L + pretreated lignin 0.593 g/L + alkali lignin 4.41 g/L) after 168 h (a), and 89 h (b).


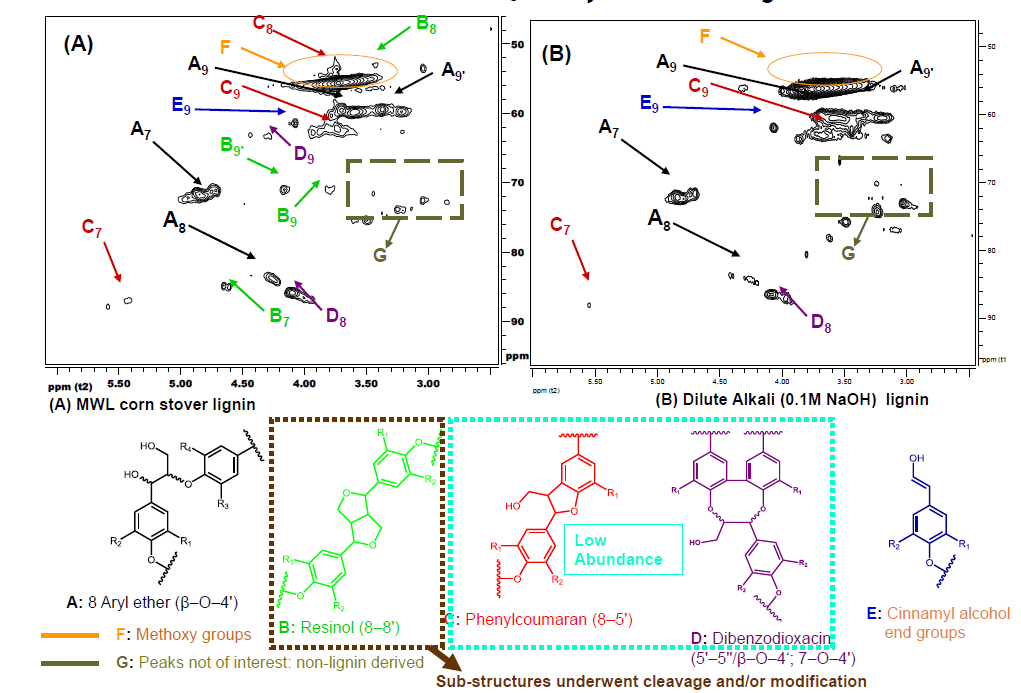


Figure S3. Gradient Selected 2D HSQC Analysis of Alkali lignin.


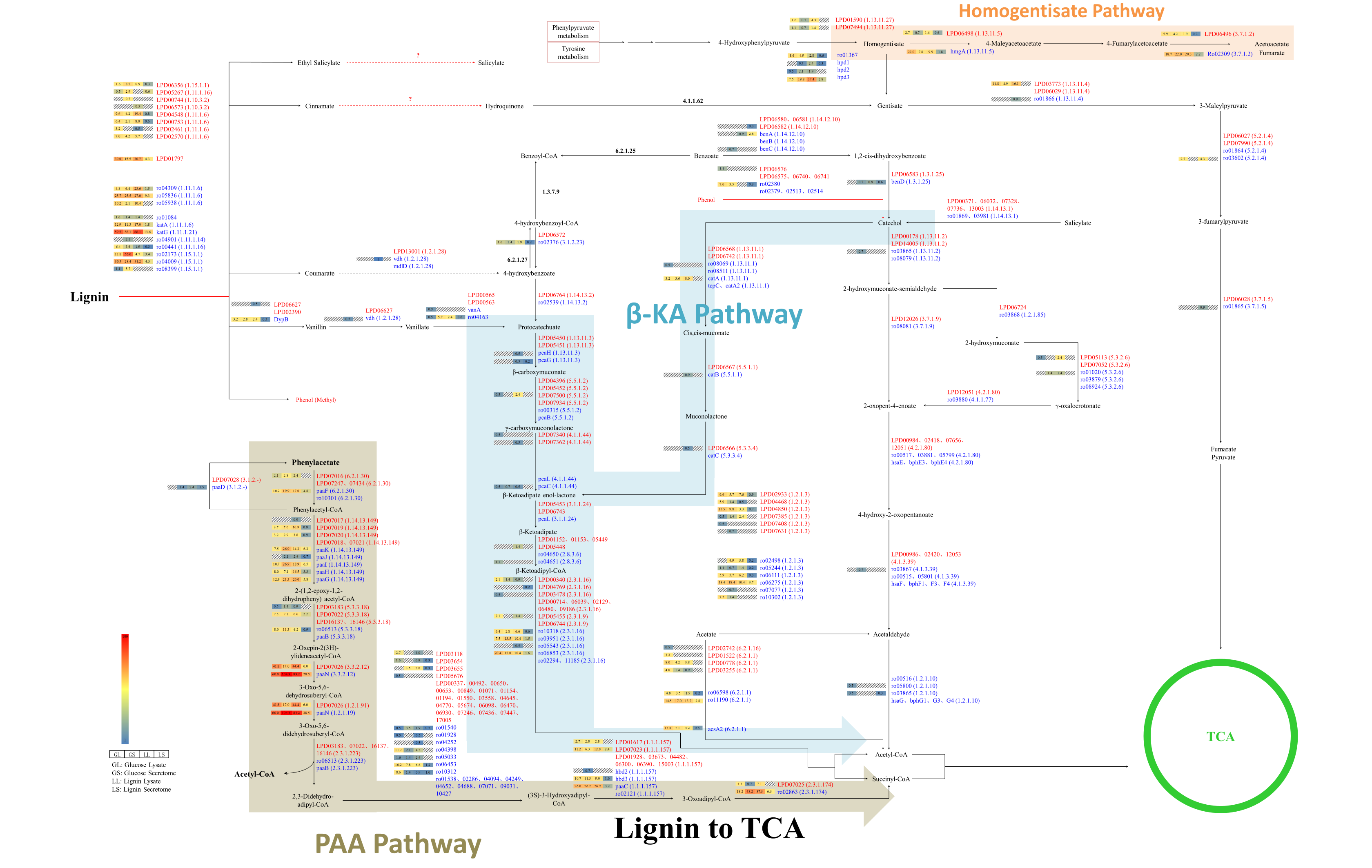


Figure S4. Proposed detailed pathways of lignin degradation in *Rhodococci*.


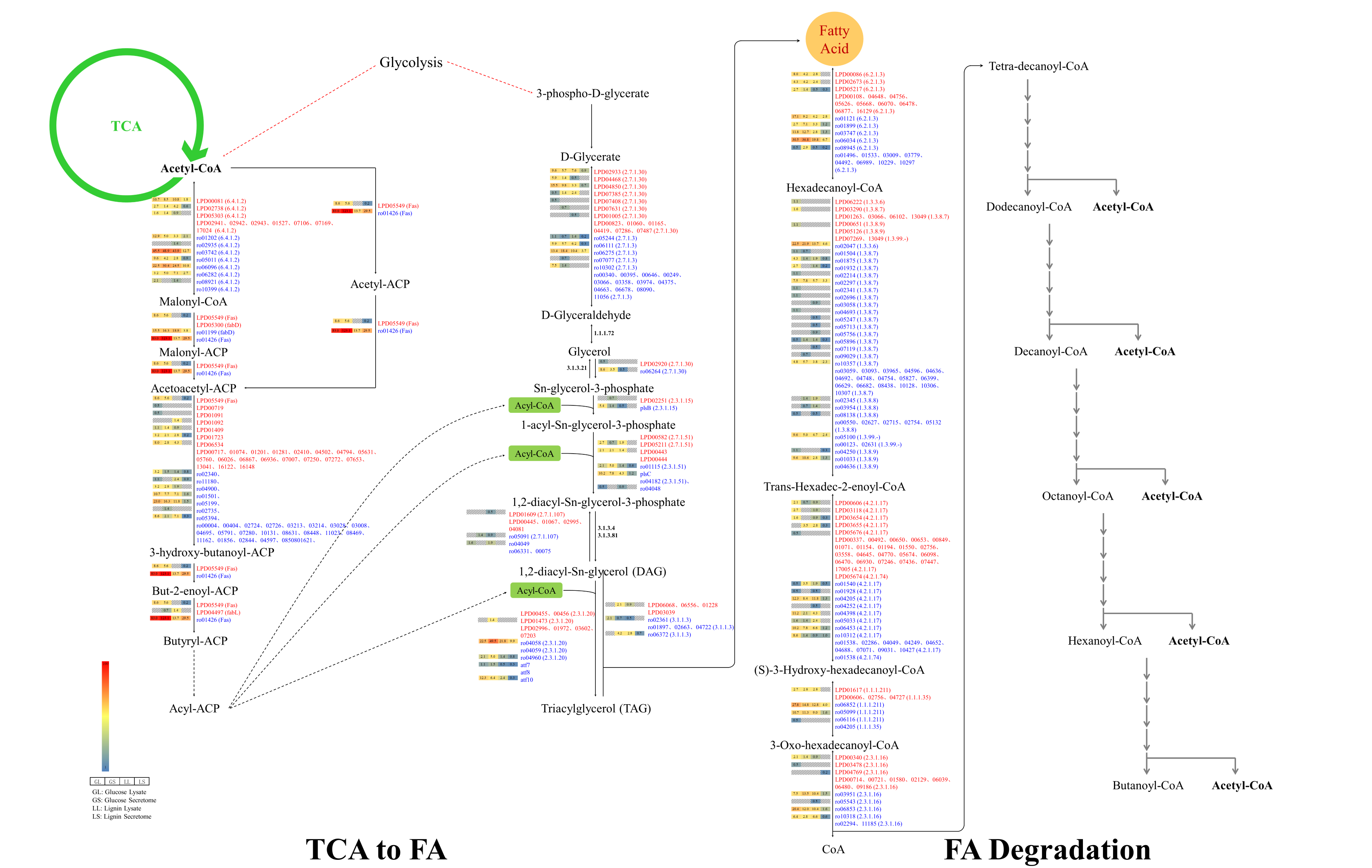


Figure S5. Proposed detailed pathways of fatty acid metabolism in *Rhodococci*.
