# Supplementary material for: Discovery of potential pathways for biological conversion of poplar wood into lipids by co-fermentation of Rhodococci strains
Source: Biotechnol Biofuels. 2019 Mar 19;12:60. doi: 10.1186/s13068-019-1395-x (PMC6423811; doi:10.1186/s13068-019-1395-x)
Supplement: Supplementary file 3 — Additional file 3. A list of abbreviations is included. [file 13068_2019_1395_MOESM3_ESM.docx]

**Abbreviations**

| **Abbreviation** | **Protein name** |
| --- | --- |
| PDH | Pyruvate dehydrogenase |
| FBP | Fructose-bisphosphate aldolase |
| PPO | 3-phenylpropionate/trans-cinnamate dioxygenase |
| SOX | Sarcosine oxidase |
| HPPD | 4-hydroxyphenylpyruvate dioxygenase |
| BENE | Benzoate transport |
| C1,2O | Catechol 1,2-dioxygenase |
| PAAA | Ring-1,2-phenylacetyl-CoA epoxidase subunit PaaA |
| PAAB | Ring-1,2-phenylacetyl-CoA epoxidase subunit PaaB |
| PAAC | Ring-1,2-phenylacetyl-CoA epoxidase subunit PaaC |
| PAAE | Ring-1,2-phenylacetyl-CoA epoxidase subunit PaaE |
| OEH | 2-keto-4-pentenoate hydratase |
| PUO | Putrescine oxidase |
| DECR | 2,4-dienoyl-CoA reductase |
| ALDH | aldehyde dehydrogenase |
| ACPD | Acyl-[acyl-carrier-protein] desaturase |
| HSP | Hsp20 family protein |
| TRX | Thioredoxin |
| TR | Thioredoxin reductase (NADPH) |
| CAT | Catalase |
| OSMC | Osmotically inducible protein OsmC |
| FAD/FMN-DH | FAD/FMN-containing dehydrogenase |
| TRES | trehalose synthase |
| 6PGD | 6-phosphogluconate dehydratase |
| XLK | xylulokinase |
| ADH | alcohol dehydrogenase |
| GPAM | glycerol-3-phosphate acyltransferase |
| GT | 4-alpha-glucanotransferase |
| WCAK | Polysaccharide pyruvyl transferase family protein WcaK |
| GB | 1,4-alpha-glucan branching enzyme |
| BC | Biotin carboxylase 1 |
| PC | Pyruvate carboxylase |
| DHAK | homodimeric dihydroxyacetone kinase |
| SPPL | starch phosphorylase |
| AGPAT | 1-acyl-sn-glycerol-3-phosphate acyltransferase |
| GDP | glycerophosphoryl diester phosphodiesterase |
| IBH | HAD-superfamily subfamily IB hydrolase, TIGR01490 |
| LCACS | long-chain acyl-CoA synthetase |
| ACC | acetyl-CoA/propionyl-CoA carboxylase carboxyl transferase subunit |
| PSD | phosphatidylserine decarboxylase |
| ACPS | 3-oxoacyl-[acyl-carrier-protein] synthase-3 |
| FADD | Long-chain-fatty-acid--CoA ligase FadD15 |
| GST | putative glutathione S-transferase |
| ECH | enoyl-CoA hydratase |
