# Supplementary material for: Discovery of potential pathways for biological conversion of poplar wood into lipids by co-fermentation of Rhodococci strains
Source: Biotechnol Biofuels. 2019 Mar 19;12:60. doi: 10.1186/s13068-019-1395-x (PMC6423811; doi:10.1186/s13068-019-1395-x)
Supplement: Supplementary file 4 — Additional file 4: Table S2. Possible lignin-degrading related enzymes in the secretome of Rhodococci during lignin fermentation. Table S3. Oxidases in the secretome of Rhodococci during lignin fermentation. [file 13068_2019_1395_MOESM4_ESM.docx]

Table S2. Possible lignin-degrading related enzymes in the secretome of *Rhodococci* during lignin fermentation.

| **NCBI Gene Symbol** | **Protein name** |
| --- | --- |
| **RHA1_RS11765** | Putative iron-dependent peroxidase (Dye-decoloring peroxidase B) |
| **RHA1_RS25800** | Catalase-peroxidase |
| **Pd630_LPD01797** | Catalase-peroxidase |
| **RHA1_RS23555** | Glutathione peroxidase |
| **RHA1_RS19475** | Superoxide dismutase, Fe-Mn family |
| **RHA1_RS20925** | Multicopper oxidase with three cupredoxin domains |

Table S3. Oxidases in the secretome of *Rhodococci* during lignin fermentation.

| **NCBI Gene Symbol** | **Protein name** |
| --- | --- |
| **RHA1_RS29685** | Glycolate oxidase |
| **RHA1_RS27355** | Putrescine oxidase |
| **Pd630_LPD02204** | Putrescine oxidase |
| **RHA1_RS08760** | Choline oxidase |
| **RHA1_RS08845** | Sarcosine oxidase subunit alpha |
| **Pd630_LPD05984** | Sarcosine oxidase subunit alpha |
| **RHA1_RS08835** | Sarcosine oxidase subunit beta |
| **RHA1_RS30305** | Cholesterol oxidase |
| **RHA1_RS03850** | Nitroalkane oxidase |
| **RHA1_RS27310** | Primary-amine oxidase |
| **RHA1_RS21975** | Urate oxidase |
| **RHA1_RS22570** | L-2-hydroxyglutarate oxidase LhgO |
