# Supplementary material for: Discovery of potential pathways for biological conversion of poplar wood into lipids by co-fermentation of Rhodococci strains
Source: Biotechnol Biofuels. 2019 Mar 19;12:60. doi: 10.1186/s13068-019-1395-x (PMC6423811; doi:10.1186/s13068-019-1395-x)
Supplement: Supplementary file 5 — Additional file 5. Supplemental information of methods is included. [file 13068_2019_1395_MOESM5_ESM.docx]

**Methods**

**Flowthrough reactor setup.** The flowthrough tubular reactor was 1.3 cm i.d. × 15.2 cm long (whose internal volume was 20.2 mL). It was constructed of 316 stainless steel parts using Vacuum Coupling Radius Seal (VCR) fittings, including one VCR male union (1.3 cm), two gasket filters (average pore size 5 μm), two VCR glands (1.3 cm × 1.3 cm), two VCR nuts, and two VCR reducing fittings (1.3 cm × 0.3 cm), obtained from Swagelok Co., Richland, WA, USA. A high-pressure pump (Acuflow Series III Pumps, Fisher, PA, USA) with a flow rate range of 0 to 100 mL/minute was used to pump the 0.05% (w/w) sulfuric acid passing through the preheating coil (0.6 cm o.d. × 0.1 cm wall, stainless steel), then the reactor and the cooling coil (0.3 cm o.d. × 0.1 cm wall). A pressure gauge (pressure range 0 to 1500 psi; Cole-Parmer Instrument Co., IL, USA) and a back-pressure-regulator (Valve and Fitting Co., WA, USA) were used to control the pressure of the flowthrough system. The reactors were heated to the target temperature in a 4-kW fluidized sand bath (model SBL-2D, Omega engineering, Inc., CT). The reaction temperature was controlled by a thermal monitor combined with a 0.3-cm stainless steel thermocouple (Omega Engineering Co., Stamford, CT) at the outlet of the flow reactor.

**Sugar analysis.** A Water HPLC system (model 2695) equipped with a refractive index detector was employed to analyze glucose in hydrolyzates of pretreatment and enzymatic hydrolysis, as well as in fermentation broth [1]. Bio-Rad Aminex HPX-87P column (Bio-Rad Laboratories, Hercules, CA, USA) was used in the HPLC system at 80 °C. HPLC grade water was used as mobile phase at flow rate of 0.6 mL/min. A series of sugar samples of known concentrations was used to build the standard curve.

**Ammonium determination.** Ammonium concentration was determined using phenol-hypochlorite method [2]. First, samples were diluted to 10 mL using deionized water. 0.4 mL of 10% (w/v) phenol solution in ethanol and 0.4 mL of 0.5% (w/v) nitroprusside solution in water was added to the samples. 1 mL of mixed solution (4:1, v/v) of 20% (w/v) trisodium citrate and 1% (w/v) sodium hydroxide solution 5%, mixed with 5% sodium hypochlorite was added to each sample. Samples were then incubated for 2 h in the dark at 37 °C. Absorbance was read at 630 nm using a UV/vis spectrophotometer (UV-2550PC, Shimadzu, Japan). Ammonium concentration was calculated using a standard curve of (NH_4_)_2_SO_4_ solution of known concentrations.

**Metabolites determination during fermentation.** The lignin degradation metabolites in the fermentation broth were determined by gas chromatography-mass spectroscopy (GC-MS). 30 mL of ethyl acetate was added to 15 mL of fermentation broth in a 50 mL centrifuge tube and was vortexed for 5 min at room temperature. The ethyl acetate layer was collected and vortexed with 20 mL of ethyl acetate for 5 min in a centrifuge tube. The collected ethyl acetate layer was placed in a rotary evaporator (Heidolph, IL, USA) for 15 min in a water bath at 30 °C. These samples were re-suspended by dissolving in 1.5 mL ethyl acetate and then transferred to GC-MS auto-injection vials. GC-MS analysis was performed on Ultra GC-DSQ (Thermo Electron, MA, USA) using electron impact ionization. Rxi-5 ms was used as the gas chromatographic column (60 m length, 0.25 mm i.d. and 0.25 μm ﬁlm thickness, Restek, PA, USA). Helium was used as the carrier gas at a constant ﬂow of 1.5 mL/min. The injection volume was 1 μL and in the splitless mode. The oven temperature was maintained at 50 °C for 5 min and raised to 320 °C at 20 °C /min. Mass spectrometer was operated in full scan mode.

**Lignin concentration analysis by Prussian blue assay.** To determine the concentration of (soluble and insoluble) lignin in the fermentation broth, 10 M NaOH was added to adjust the pH of the samples to 12.5 to dissolve lignin. 1.5 mL of the samples was transferred into testing tubes. 100 μL of 8 mM K_3_Fe(CN)_6_ was added into the tube. Then, 100 μL of 0.1 M FeCl_3_ was added immediately. The samples were shake for 5 minutes to mix well followed by transferring to 1 cm cuvettes to obtain the absorbance at 700 nm by a UV/vis spectrophotometer (UV 2550PC, Shimadzu, Japan). Standard curve was based on same method with known concentration of lignin. Triplicates were carried out in this experiment. Total lignin degradation was calculated by the equation below:

$$Lignin degradation \left( \% \right)=(1-\frac{lignin concentration after fermentation}{initial lignin concentration before fermentation})\times100$$

**References**

1. Sluiter A, Hames B, Ruiz R, Scarlata C, Sluiter J, Templeton D: **Determination of sugars, byproducts, and degradation products in liquid fraction process samples**. *Golden: National Renewable Energy Laboratory* 2006.

2. Weatherburn M: **Phenol-hypochlorite reaction for determination of ammonia**. *Analytical chemistry* 1967, **39**(8):971-974.
